# Rapid Cytoskeletal Response of Epithelial Cells to Force Generation by Type IV Pili

**DOI:** 10.1371/journal.pone.0017088

**Published:** 2011-02-14

**Authors:** Dirk Opitz, Berenike Maier

**Affiliations:** Institut für Molekulare Zellbiologie, Westfälische Wilhelms Universität, Münster, Germany; Weill Cornell Medical College of Cornell University, United States of America

## Abstract

Many bacterial pathogens interfere with cellular functions including phagocytosis and barrier integrity. The human pathogen *Neissieria gonorrhoeae* generates grappling hooks for adhesion, spreading, and induction of signal cascades that lead to formation cortical plaques containing f-actin and ezrin. It is unclear whether high mechanical forces generated by type IV pili (T4P) are a direct signal that leads to cytoskeletal rearrangements and at which time scale the cytoskeletal response occurs. Here we used laser tweezers to mimic type IV pilus mediated force generation by T4P-coated beads on the order of 100pN. We found that actin-EGFP and ezrin-EGFP accumulated below pilus-coated beads when force was applied. Within 2 min, accumulation significantly exceeded controls without force or without pili, demonstrating that T4P-generated force rapidly induces accumulation of plaque proteins. This finding adds mechanical force to the many strategies by which bacteria modulate the host cell cytoskeleton.

## Introduction

Various bacterial pathogens interfere with signalling pathways of their host cells for example to increase or decrease the probability for phagocytosis [1], [2], to enable intracellular movement [3], or to break cellular barriers by modulating intercellular junctions [4]. The human pathogens *N. gonorrhoeae* (gonococcus) and *Neisseria meningiditis* reprogram functions in epithelial and endothelial cells for transversal of the respective barriers [1], [5]. However, the use of secretion systems to inject effectors into their host cells has not been demonstrated so far. Type IV pili (T4P) are important for initial adhesion and virulence [5]. These extra-cellular polymeric filaments act as molecular grappling hooks that elongate by polymerization, adhere to surfaces, and retract by depolymerization [6], [7]. During retraction, mechanical force exceeding 100 pN is generated [8]. Tension on the surface is tunable through force-induced pilus elongation around 100 pN [9], [10], [11]. T4P form bundles which generate forces in the nanonewton range [12]. Force generation by individual T4P is slightly reduced to 75 pN during infection of epithelial cells by gonococci [13].

It is conceivable that T4P retraction acts as a mechanical signal to epithelial cells leading to reorganization of the cytoskeleton. By comparison of infection by wild type gonococci with a retraction-deficient (*pilT-*) strain, it has been demonstrated that the formation of cortical plaques containing f-actin, transmembrane proteins, and membrane-cytoskeleton linkers such as ezrin depends on T4P retraction [14], [15]. Furthermore, mechanosensitive and cytoprotective pathways were activated by wild type cells but not by retraction-deficient strains [16]. By generating force on the order of 4 pN on T4P-coated magnetic beads and BSA-coated magnetic beads, force-dependent activation of the MAPK pathway and accumulation of f-actin were demonstrated after several hours, but the response was not specific to T4P [16], indicating that epithelial cells detect type IV pilus retraction at a time scale of several hours by regulating gene expression. However, mammalian cells sense mechanical force and rigidity and they respond at very different time scales. Recent reports demonstrate Src activation [17] at the subsecond scale, Ca^2+^ influx [18] and formation of focal complexes at the time scale of minutes [19], and cellular differentiation at a time scale of hours [20]. Sensing mechanisms potentially include cryptic binding sites which open upon stretch, opening of mechanosensitive channels, or controlling membrane curvature [21].

Thus the time scale of mechanosensing correlates different mechanisms. It is unclear, at which time post infection by gonococci mechanosensing occurs and whether mechanosensitive pathways during gonococcal infection are specific to T4P. To investigate whether T4P-generated force during gonococcal infection leads to accumulation of host cell proteins, we designed a laser-tweezers based assay. This assay enabled us to quantify the force dependent dynamics of cytoskeletal proteins within seconds of force application.

## Results

### Type IV pilus-coated beads bind rapidly and irreversibly to Hec1B epithelial cells

**Figure 1:**
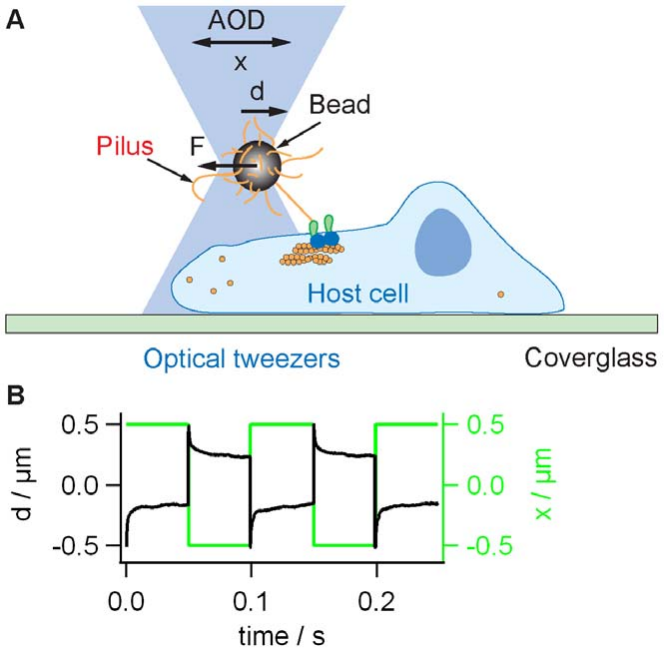
Experimental setup for “infection” with T4P-coated beads. A) Sketch of the setup. Beads were trapped and placed onto the surface of Hec1B cells. When the center of the laser trap is actively moved by *x*, the deflection of the bead from the center of the trap *d* is proportional to the force *F*. B) Green line: The laser focus was moved by x  =  ±0.5 µm with respect to the initial position with a frequency of 10 Hz. Black line: Typical signal of the quadrant photodiode showing the position *d*. Deflection from 0 verifies binding of the bead to the surfaces of the epithelial cell.

We integrated a movable optical trap into a confocal microscope (Fig. 1A). 2 µm latex beads were coated with T4P preparation. Coating of the beads with pili was verified using immunofluorescence (Figure S1). Coated beads were trapped in the laser trap and approached to the surface of Hec1B cells. The stiffness of the laser trap was 0.25 pN/nm, thus the maximum force applied to one bead was on the order of 100pN. Wild type gonococci generate on average seven T4P around the contour of the cell body and we estimate that approximately 2–3 T4P retract simultaneously in one cell [22]. Thus the bacteria most likely exert force pulses to their host cell. Please note that we intend to simulate a different scenario as expected for cellular adhesion to extracellular matrix where the turn-over of attachment occurs at a slower time scale. The length of individual pili is approximately 1 µm and the velocity of retraction is 2 µm/s [10]. Therefore, we moved the beads by ±0.5 µm using a step-function and with a frequency of 10 Hz. Similar to the situation of infection, T4P-coated beads generated force only when they were firmly attached to the surface of the epithelial cell. We monitored the attachment of the beads as the response of a quadrant diode to bead movement (see Methods). The bead only generated a signal if it was attached to the cell surface. Since the force generated by the bead is proportional to the deflection, the force was highest if the bead was firmly attached. We found that the beads attached very rapidly and irreversibly to the surface (Fig. 1B, C). An initial rapid deflection of the bead from the center of the trap was followed by relaxation, i.e. the bead moved slowly back towards the center of the trap. Although the temporal development of the deflection was reproducible, the relaxation times were highly variable throughout an experiment and between experiments. Thus we can conclude that with our assay we applied varying forces in the range between 30–100 pN. In rare cases, the epithelial cell pulled actively on the cell and displaced the beads significantly from its initial position; these events were disregarded.

### Dynamics of actin-EGFP and ezrin-EGFP accumulation

**Figure 2:**
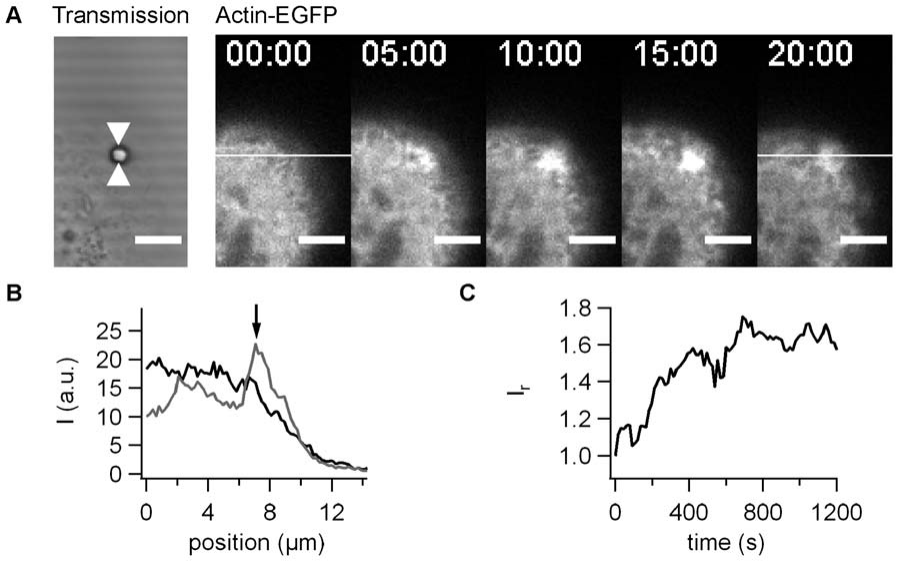
Dynamics of actin-EGFP accumulation with pilus coated beads. A) One confocal plane of actin-EGFP producing epithelial cells was monitored during 20 min. Scale bar 5 µm. B) Intensity plot through the ROI shown in A) at black: 0 min, grey: 20 min post adhesion. C) *I_r_* as a function of time for the time lapse in A).

Actin has been shown to accumulate strongly in cortical plaques [14]. Thus, we monitored Hec1B cells expressing actin-EGFP. During bead movement, we continuously recorded one confocal plane (Fig. 2A).

We found that actin-EGFP accumulated within minutes of force application (Fig. 2A, B, Movie S1). In rare cases (<8%) we observed the formation of bleb-like membrane protrusions and we therefore discarded these cells from our analysis. To quantify the accumulation, for each pixel we calculated the ratio between the intensity and the average intensity in a surrounding area with a radius of 4 µm. This relative fluorescence intensity *I_r_* was then averaged within a circular area with the radius of 2 µm around the bead (see Methods). We found that *I_r_* increased without delay from the time when we started to apply force (Fig. 2C).

**Figure 3:**
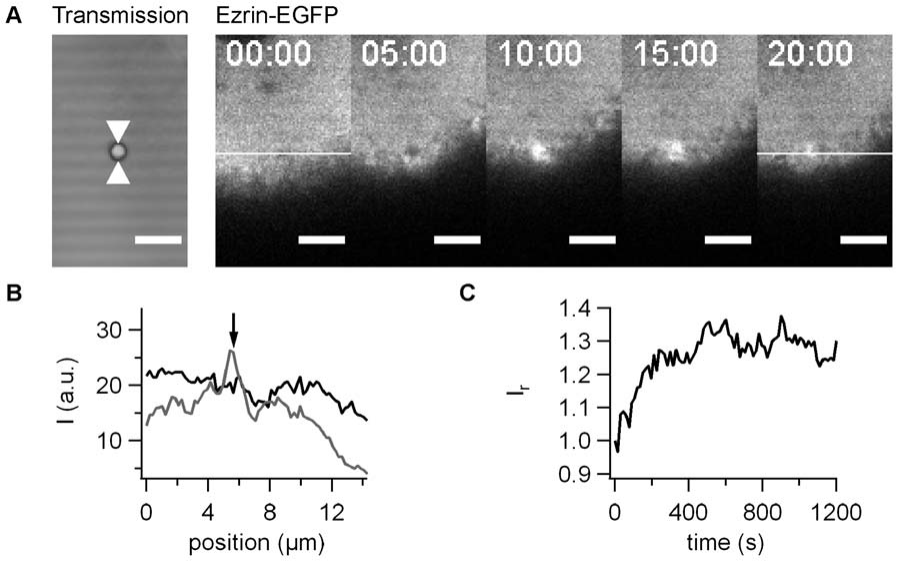
Dynamics of ezrin-EGFP accumulation with pilus coated beads. A) One confocal plane of ezrin-EGFP producing epithelial cells was monitored during 20 min. Scale bar 5 µm. B) Intensity plot through the ROI shown in A) at black: 0 min, grey: 20 min post adhesion. C) *I_r_* as a function of time for the time lapse in A).

To verify that protein accumulation upon force application was not specific to actin, we monitored the behavior of ezrin-EGFP under the same conditions. Similar to actin-EGFP, ezrin-EGFP started to accumulate immediately (Fig. 3A,C, Movie S2) and strong accumulation was observed after 20 min (Fig. 3A, B). Thus we established accumulation of two different cytoskeletal proteins upon force application on T4P-coated beads.

### Accumulation of actin-EGFP and ezrin-EGFP depends on force

**Figure 4:**
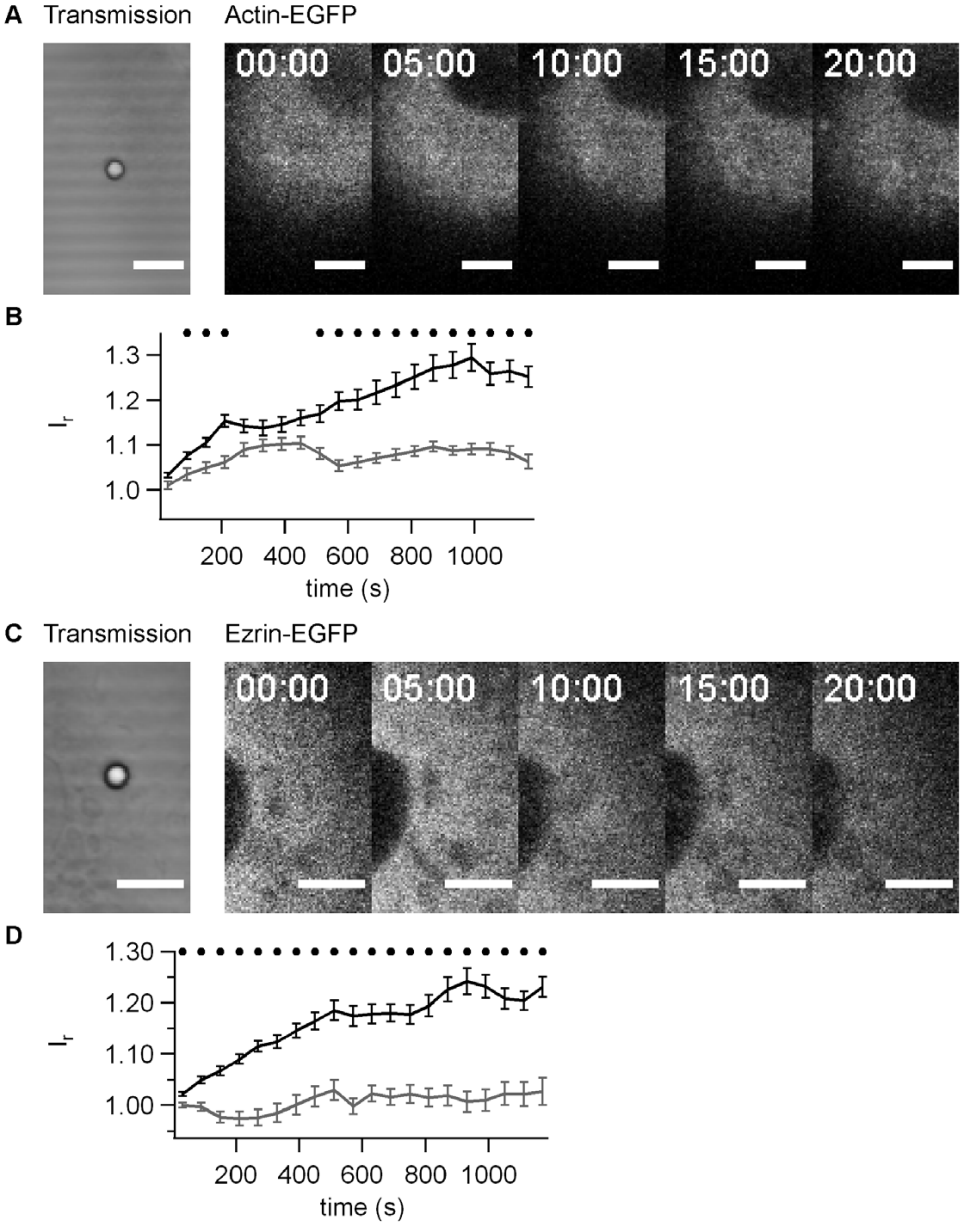
Accumulation of actin-EGFP and ezrin-EGFP depends on force. A) Typical time lapse for actin-EGFP for beads without force application by laser tweezers. B) Relative fluorescence intensity *I_r_* as a function of time for actin-EGFP averaged over 20 beads. C) Typical time lapse for ezrin-EGFP for beads without force application by laser tweezers. D) Relative fluorescence intensity *I_r_* as a function of time for ezrin-EGFP averaged over 30 beads. Black line: application of F ≤100 pN, grey line: F = 0. Black dots mark statistical significant different values with force and without force (t-Test significance level p<0.01)

We addressed the question whether the accumulation of actin and ezrin was dependent on the force applied to the bead. Beads on the same cell that were not subject to force application were not associated with increased fluorescence intensity by either actin-EFGP (Fig. 4A) or ezrin-EGFP (Fig. 4C). We compared the average fluorescence intensity as a function of time from multiple experiments and found a significantly higher accumulation in response to force application already within 150 s for actin-EGFP (Fig. 4B) and within 30 s for ezrin-EGFP (Fig. 4D). The accumulation persisted for at least 20 min. This experiment clearly shows that both actin and ezrin accumulate in response to force generated through bead movement.

### Accumulation of actin-EGFP and ezrin-EGFP depends on type IV pili

**Figure 5:**
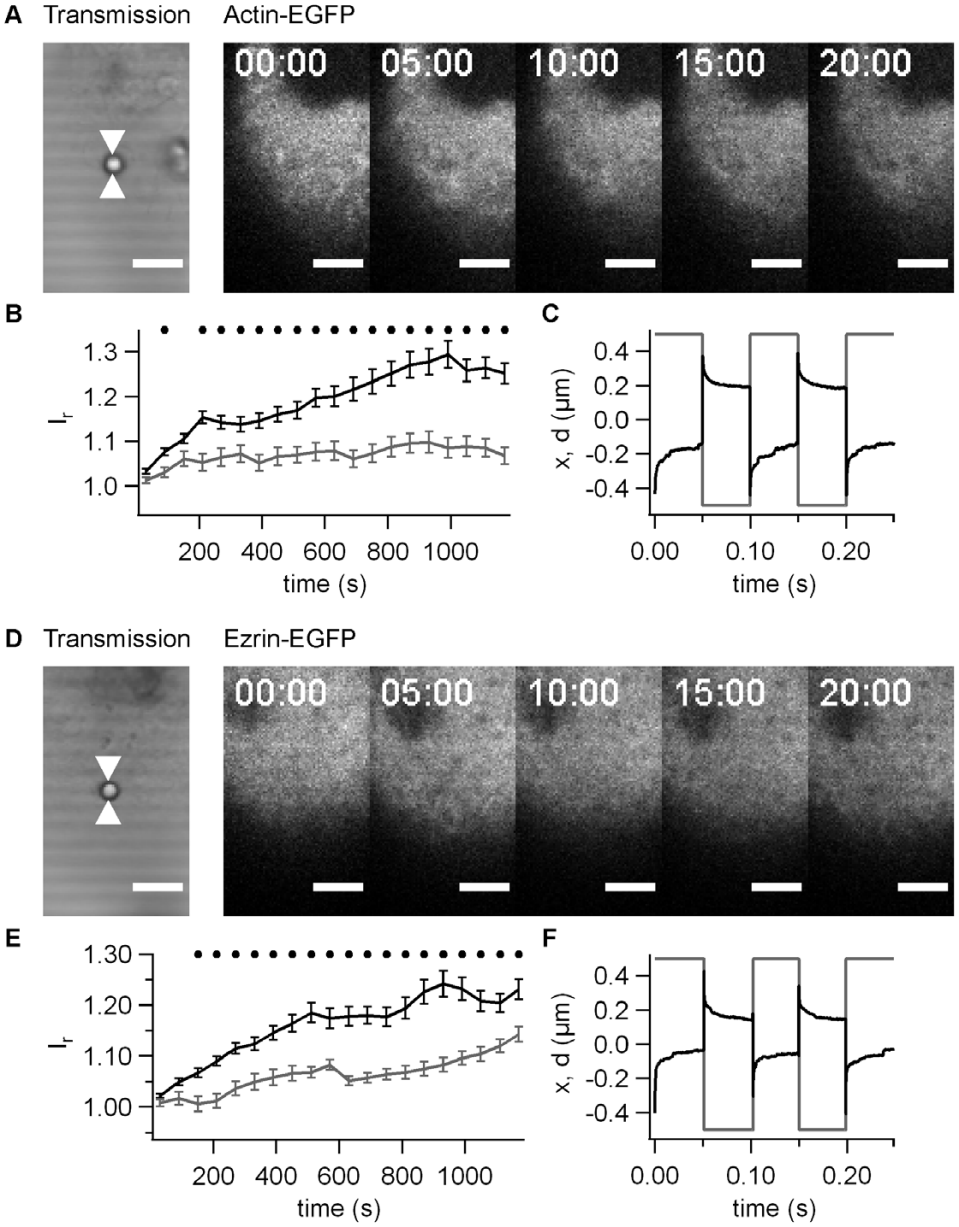
Accumulation of actin-EGFP and ezrin-EGFP depends on T4P. A) Typical time lapse for actin-EGFP for beads coated with *pilE-* preparation (no pili). B) Relative fluorescence intensity *I_r_* as a function of time for actin-EGFP averaged over 15 beads. Black line: T4P preparation from N400 wt pili, grey line: T4P preparation from *pilE-* strain. C) Grey line: The laser focus was moved by x  =  ±0.5 µm with respect to the initial position with a frequency of 10 Hz. Black line: Typical signal of the quadrant photodiode showing the position *d*. D) Typical time lapse for ezrin-EGFP for beads coated with *pilE-* preparation. E) Relative fluorescence intensity *I_r_* as a function of time for ezrin-EGFP averaged over 15 beads. Black line: T4P preparation from N400 wt pili, grey line: T4P preparation from *pilE-* strain. F) Grey line: The laser focus was moved by x  =  ±0.5 µm with respect to the initial position with a frequency of 10 Hz. Black line: Typical signal of the quadrant photodiode showing the position *d*. B,D) Black dots mark statistical significant different values with pili and without pili (t-Test significance level p<0.01).

**Figure 6:**
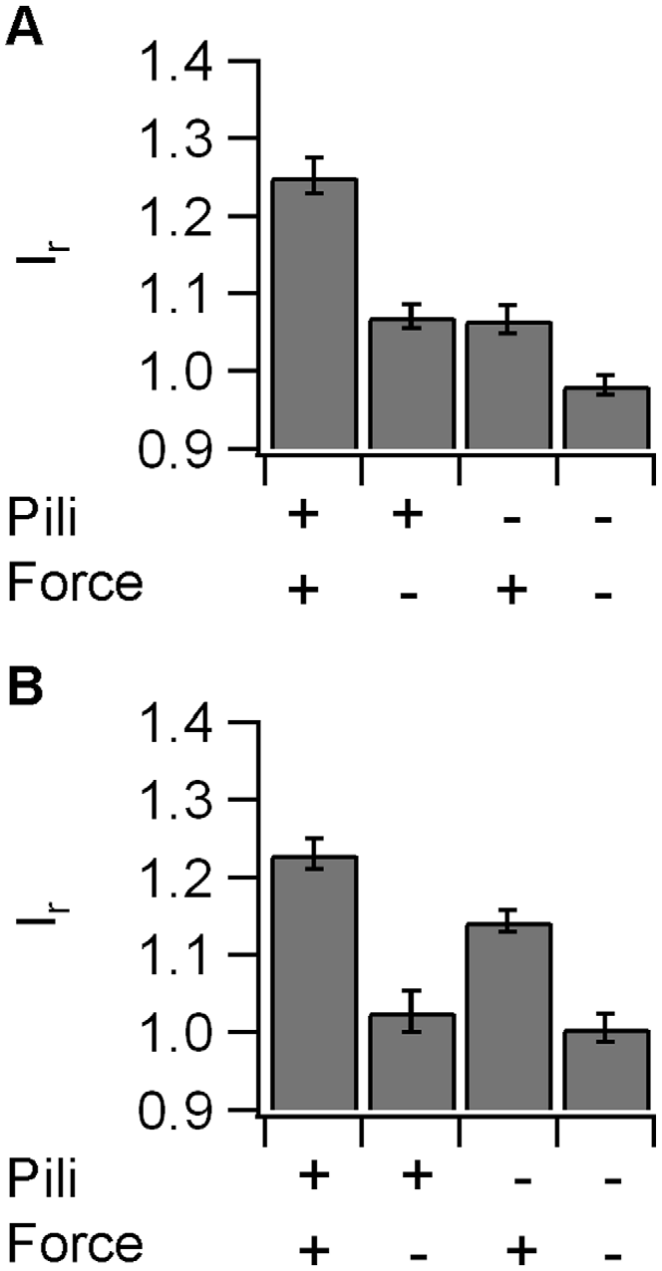
Accumulation of actin and ezrin after 20 min of force application. Average relative fluorescence intensity *I_r_* after 20 min of force application for A) actin-EGFP and B) ezrin-EGFP.

So far, we have shown that force is essential for ezrin and actin accumulation. However, it was still unclear, whether T4P were necessary, i.e. whether the cytoskeletal response was specific to type IV pili. Since the T4P preparation is not very pure, we used the same preparation protocol using a *pilE-* strain that did not express the major pilin subunit and did not generate T4P. These beads attached rapidly and irreversibly to the cell surface and we verified that force was applied on the epithelial cell through the bead by checking the elastic response. When the laser trap was moved from −0.5 µm to +0.5 µm, the bead followed rapidly indicating that force on the order of 30–100 pN was applied (Fig. 5C, F). We found no significant accumulation of actin-EGFP (Fig. 5A, B) and strongly delayed accumulation of ezrin-EGFP (Fig. 5D, E). The accumulation was significantly higher in response to T4P-coated beads within 90 s for actin-EGFP (Fig. 5B) and within 150 s for ezrin-EGFP (Fig. 5F). The accumulation persisted for at least 20 min (Fig. 6A,B) and although the ezrin-EGFP accumulated slightly within this time frame, accumulation using pilus-coated beads was significantly higher at this time point (Fig. 6B). We conclude therefore, that mechanical force generated by T4P elicits accumulation of actin and ezrin within minutes.

## Discussion

### “Infection” with T4P-coated beads

We developed an assay that is highly suitable for elucidating the effect of T4P and force generation on the dynamics of epithelial cells during 20 min post infection. Since the bacterial cell body was replaced by a bead, the participation of bacterial molecules other than T4P pili (or proteins presented through attachment to the pilus) in eliciting epithelial cell response can be excluded. In addition, residual interactions caused by impurities in the T4P preparation can be excluded by comparison with *pilE-* preparations. Furthermore, both amplitude and maximum force generated can be controlled. Using the laser tweezers assay, the time point of adhesion is tightly controlled and therefore the response in the range of seconds can be analyzed. Accordingly, we detected a response as early as 30 sec whereas previous reports stated a response within 15 min post infection [23]. This discrepancy is most likely due to uncontrolled adsorption of bacteria to the epithelial cell. Howie *et al* have previously used a magnetic assay and found that force generation with a maximum of 4 pN by beads adhered to epithelium cells influenced gene expression after two hours [16]. However, the response was indistinguishable from control beads coated with BSA. Two factors are highly likely to explain the discrepancy. First, the force was by an order of magnitude lower than the force applied in this study and by a factor of 20 lower than the force generated by a single pilus during infection of epithelial cells. Second, we used polyethlyen glycol rather than BSA for passivating the beads in order to inhibit unspecific interactions between the bead and the host cell, which is most likely to be more efficient.

### Time scales of plaque formation

The accumulation of ezrin below the T4P-coated bead was significantly higher than the controls without pili or without force within 30–150 sec. Interestingly, a maximum of phosphorylation and reorganization of ezrin was observed 2–5 min after stimulation with epidermal growth factor (EGF) in A431 cells [24]. The maximum of phosphorylation was observed at 2–5 min and correlated with membrane ruffles. Thus, ezrin reorganization occurred on similar time scales in response to both force generation and EGF stimulation. With gonococcal infection, mature plaques are formed after 2–4 h post infection and the density of plaque proteins including ezrin increased during this time period (Höne & Maier, unpublished). Howie *et al* showed that T4P retraction enhanced expression of genes know to be activated in response to mechanical stress [16]. Tension-induced gene expression is unlikely to explain the accumulation of plaque proteins at the time scale observed in our experiments. Thus we suggest that rapid T4P-mediated mechanosensing leads to initial accumulation of plaque proteins which is the followed by a stronger accumulation controlled by gene regulation on a time scale of hours.

What is the molecular mechanism of mechanosensing? Laser tweezers have been used to study reinforcement of the cytoskeleton during the formation of focal adhesions using fibronectin-coated beads [19]. Studies on menigococcal infection of endothelial cells showed that an important component of focal adhesions, namely paxilin, does not accumulate below bacterial microcolonies [25], suggesting that bacteria do not trigger the formation of focal adhesions. Four mechanisms have been proposed, namely formation of catch-bonds, opening of mechano-sensitive channels, presentation of cryptic sites on enzymes and other proteins, and control of enzymatic activity [26]. Our data provide no evidence for extracellular catch-bond formation between T4P and cellular receptors, since the T4P-coated beads bind tightly to the epithelial cell surface without force application. Recent reports show that lipid rafts form in endothelial cells below microcolonies of *N. meningiditis* and that accumulation of ezrin is dependent on lipid rafts [27] and in particular on PIP_2_ [28]. Dominant negative ezrin inhibits actin accumulation [25]. Furthermore, it has been revealed that gonococcal adhesion mobilizes Ca^2+^ within minutes after adhesion in a T4P dependent manner [29]. Interestingly, both Ca^2+^ influx has been shown to be necessary for force-dependent formation of localized actin protrusions in fibroblasts which might counteract global deformation of the cell [30]. It is therefore tempting to speculate that force sensing occurs within the host cell membrane and we are confident that the assay presented here is suitable for studying the temporal sequence of the early mechanosensing in future experiments.

### Conclusion

In conclusion, we have demonstrated that epithelial cells respond to mechanical force generated through type IV pili within minutes. We propose that bacteria do not only hitch-hike chemical pathways for altering the behavior of their host cells but they also use rapid mechanical pathways. Our assay will be useful to study the temporal sequence of signaling events the lead to the formation of cortical plaques during gonococcal infection and should be applicable to other pathogens that generate T4P including *N. meningiditis*, or *Pseudomonas aeruginosa*.

## Materials and Methods

### Bacterial strains, cell culture, and media

We used the *N. gonorrhoeae* strains N400 (VD300_recA6ind_, denoted as wt strain) and VD300_PilEind_ (*pilE-*) for this study. Bacteria were maintained on agar containing 5 g L^−1^ NaCl (Roth), 4 g L^−1^ K_2_HPO_4_ (Roth), 1 g L^−1^ KH_2_PO_4_ (Roth), 15 g L^−1^ Proteose Peptone No. 3 (BD), 0.5 g L^−1^ Starch (Sigma Aldrich), 10 ml IsoVitaleX Enrichment (BD), Bactor Agar 10 g L^−1^ (BD) and were grown at 37°C and 5% CO_2_. Experiments were performed in PBS with 2 mM l-glutamine (Gibco), 8 mM sodium pyruvate (Gibco), 5 mM ascorbic acid (Roth), 30 mM HEPES (Roth) and 1 mg mL^−1^ BSA.

We used the epithelial cell line Hec1B (ATCC HTB-113). The cells were cultured in DMEM with phenol-red (Gibco) supplemented with 2 mM l-glutamine, 10% FBS (Gibco), 1 mM sodium pyruvate. Cells were transfected with either pEGFP-N1ezEGFP [31] or pEGFP-aktin [32] by electroporation with Amaxa Cell Line Nucleofactor Kit T (Lonza Group Ltd., Switzerland). Cells were grown for two days after transfection. For experiments a suspension of 2 µm coated latex beads was added, mounted on a cover slide and sealed. Each sample was used for up to two recordings of a single bead and each bead was attached to a different cell.

### Coating of beads with T4P preparations

Type IV pili were purified according to a method described by Brinton *et al.* [33]. In short, bacteria were solubilized in 150 mM ethanolamine buffer pH 10.5. T4P were sheared off by vigorous vortexing. Bacteria were centrifuged and 10% saturated ammonium sulfate was added to the supernatant. The supernatant was incubated for 30 min at room temperature and subsequently centrifuged to collect the pili. The pellet was washed in TBS pH 8.0 and stored in ddH_2_O at −20°C.

Pilus coating of microspheres was performed using the PolyLink Coupling Kit (PolyScience) with carboxylated polystyrene beads. Coated beads were stored in 10 mM Tris pH 8 with 0.05% mPEG 10′000 (Rapp Polymere).

### Confocal laser tweezers

We modified a confocal microscope (Nikon TE2000 C1) by coupling a trapping laser (J20I-BL-106C, Spectra Physics, wavelength 1064 nm) into the fluorescence port. The position of the trap in the sample was steered by a two-axis acousto-optical deflector (DTD-274HD6 Collinear Deflector, IntraAction Corp., USA) positioned in a conjugated plane to the back aperture of the 60x objective (Nikon Plan Apochromate VC 60x N.A. 1.20). The position of the bead with respect to the center of the trap was measured by a four quadrant photo diode using the back focal plane detection method [34].

Fluorescence was imaged using 488 nm laser (LASOS LGK 7880) and pinhole radius of 100 µm. Images were recorded every 10–20 s. Using a transmission detector (Nikon) we were able to visualize the sample in dia-illumination mode simultaneously to the fluorescence images. This gave us a glimpse of the bead movement during the experiments.

### Image analysis

Fluorescence images were analyzed using ImageJ. Relative fluorescence intensities were calculated by dividing the raw images with their blurred versions (Gaussian blur, radius 4 µm). Then an average of the relative intensities in an area with the radius 2 µm around the bead was measured. These values were normalized to their initial value at t  = 0 and averaged over multiple experiments.

## Supporting Information

Figure S1 Immunofluorescence (rabbit anti-PilE (Micheal Koomey) and Alexa Fluor 488 goat antirabbit, Invitrogen) of 2 µm latex beads coated with the results of crude pilus preparations of A) wildtype bacteria (N400) B) *pilE-* bacteria. C) As a control uncoated beads were used. Scale bar: 5 µm. d) Average fluorescence of single beads (error bar: standard deviation).
(JPG)

Movie S1 Dynamics of actin-EGFP accumulation with pilus coated beads (see Fig. 2). 20min time series after sliding average over four images (1min). Scale bar: 5 µm.
(AVI)

Movie S2 Dynamics of ezrin-EGFP accumulation with pilus coated beads (see Fig, 3). 20min time series after sliding average over four images (1min). Scale bar: 5 µm.
(AVI)
