# Supplementary figures and images for: Rapid Cytoskeletal Response of Epithelial Cells to Force Generation by Type IV Pili
Source: PLoS One. 2011 Feb 14;6(2):e17088. doi: 10.1371/journal.pone.0017088 (PMC3038865; doi:10.1371/journal.pone.0017088)

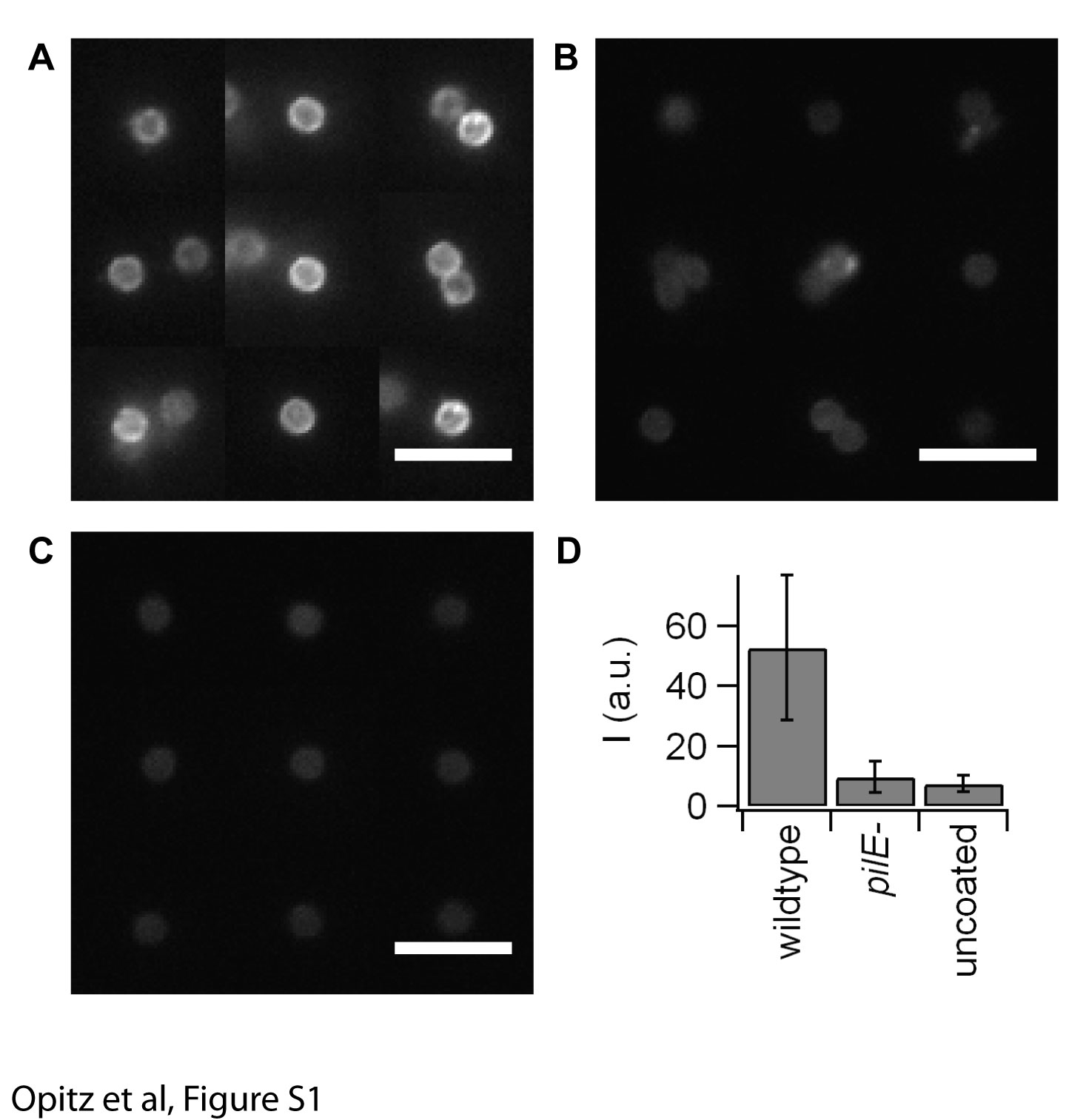

Supplement: Figure S1 — Immunofluorescence (rabbit anti-PilE (Micheal Koomey) and Alexa Fluor 488 goat antirabbit, Invitrogen) of 2 µm latex beads coated with the results of crude pilus preparations of A) wildtype bacteria (N400) B) pilE- bacteria. C) As a control uncoated beads were used. Scale bar: 5 µm. d) Average fluorescence of single beads (error bar: standard deviation). (JPG) [file pone.0017088.s001.jpg]
